# Supplementary material for: Structural basis for membrane tethering by a bacterial dynamin-like pair
Source: Nat Commun. 2018 Aug 21;9:3345. doi: 10.1038/s41467-018-05523-8 (PMC6104087; doi:10.1038/s41467-018-05523-8)
Supplement: Supplementary file 1 — Supplementary Information [file 41467_2018_5523_MOESM1_ESM.pdf]

Structural basis for membrane tethering by a bacterial dynamin-like pair

Jiwei Liu[1], Jeffrey K. Noel[2], Harry H. Low[1*]

[1]Department of Life Sciences, Imperial College, London, SW7 2AZ, UK

[2]Max Delbrück Center for Molecular Medicine, Kristallographie,

Robert-Rössle-Strasse 10, Berlin 13125, Germany

*Corresponding author:

Harry H. Low

Department of Life Sciences, Imperial College, London, SW7 2AZ, UK

Phone: +44 207 594 3064

Email: h.low@imperial.ac.uk

Supplementary Figures

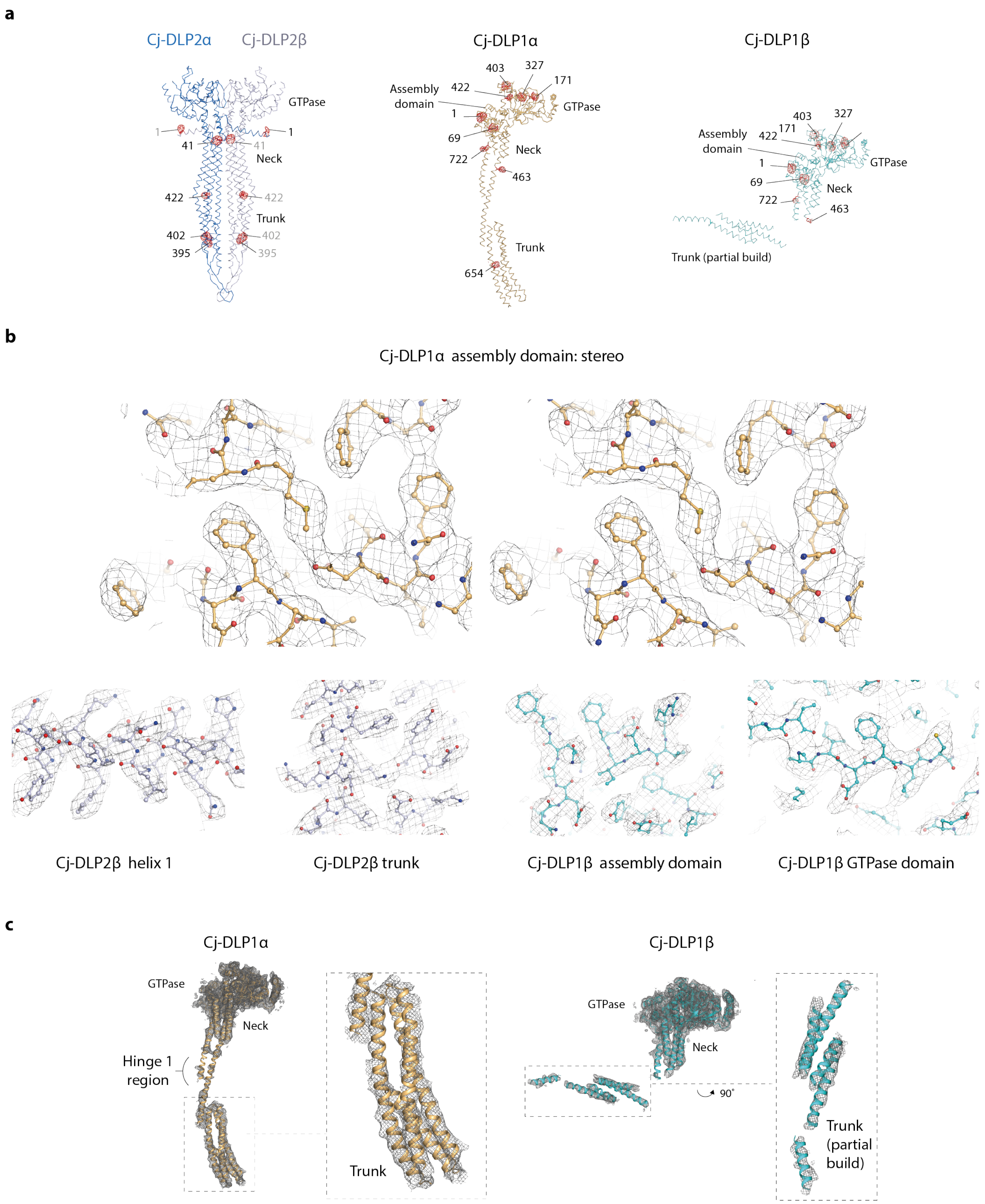

Supplementary Figure 1 | Cj-DLP1 and Cj-DLP2 sequence assignment and model quality. (a) Ribbon representation of Cj-DLP1 and Cj-DLP2 showing anomalous difference

density contoured at 3σ (red), which confirms the position of 27 from 28 seleno-methionines. (**b**) Ball and stick representation showing typical model quality with sharpened 2mFo-DFc electron density at 1σ. Side chain detail is generally resolved throughout Cj-DLP2, and Cj-DLP1 assembly, GTPase and neck domains. (**c**) Cartoon representation of Cj-DLP1 showing overall quality of unsharpened electron density contoured at 0.75σ. High flexibility within the hinge 1 region and trunk reduces map quality so that poly-alanine main chain was fitted in these regions (see Methods for full details). Selenomethionine amino acid 654 provided unequivocal identification and orientation of the Cj-DLP1$_{\alpha}$ trunk main chain.

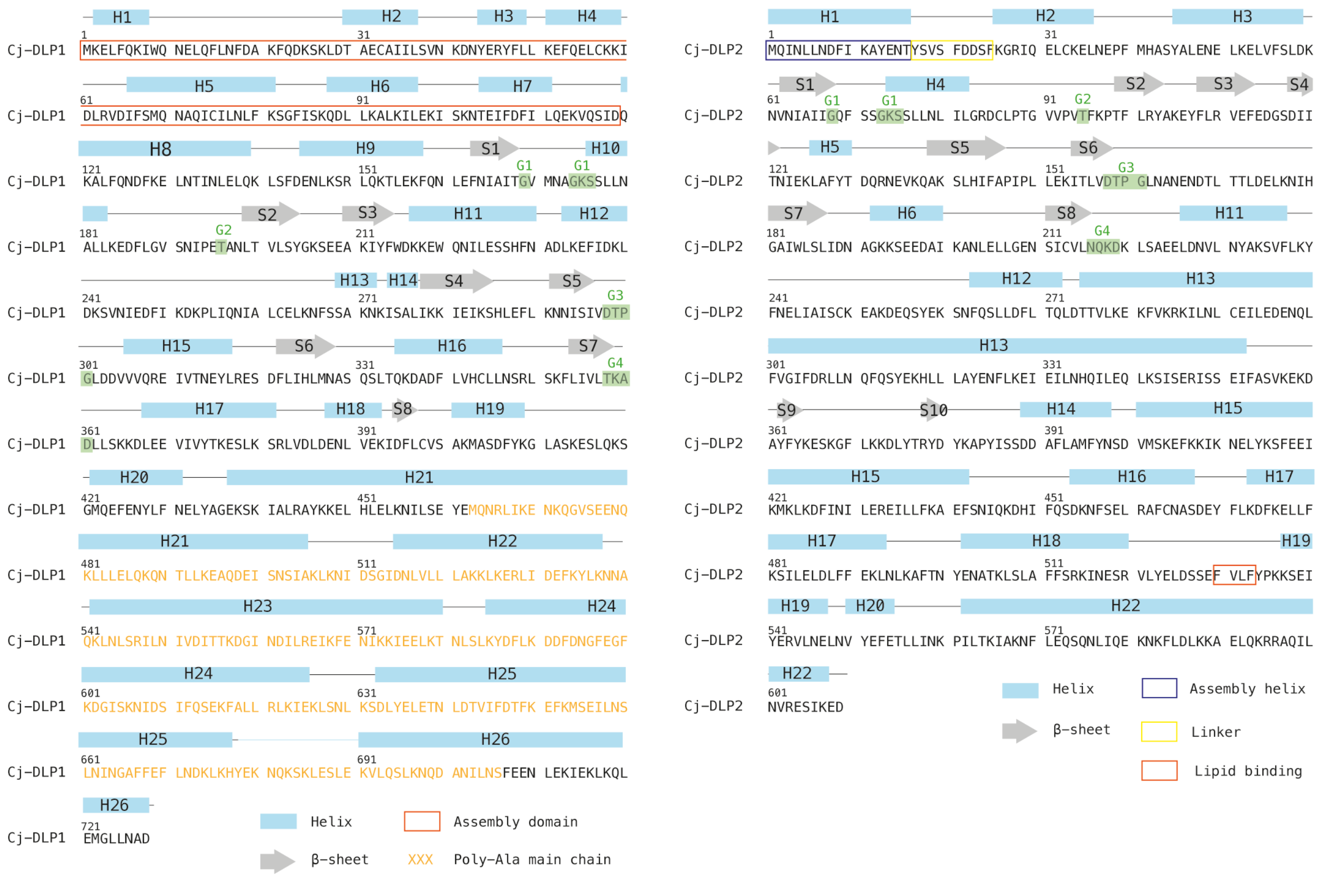

Supplementary Figure 2 | Structural assignment of Cj-DLP1 and Cj-DLP2 using DSSP [1].

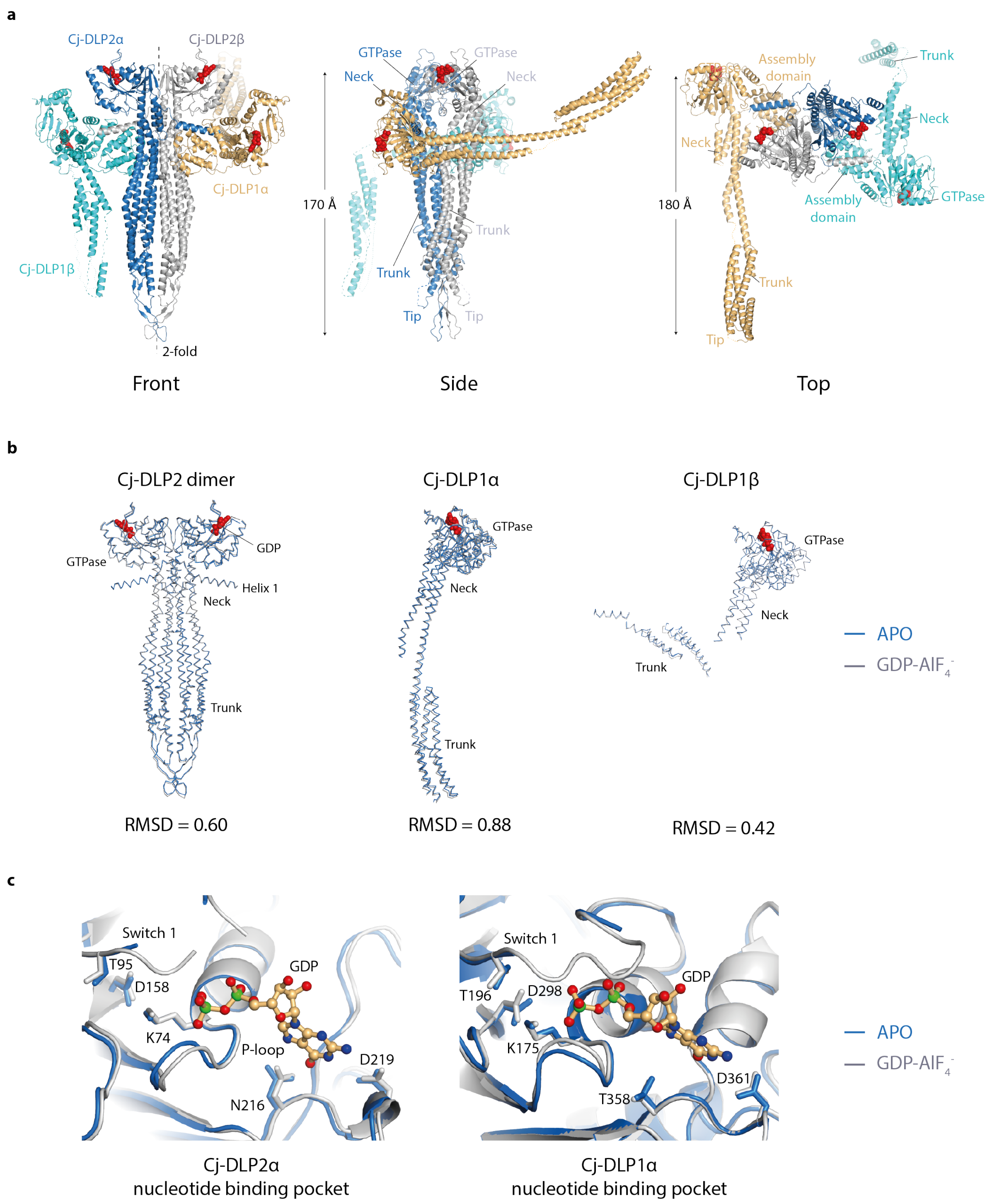

Supplementary Figure 3 | Structural comparison of Cj-DLP1/2$_{tetramer}$ apo and GDP·AlF_4^- bound models. (**a**) The crystal structure of Cj-DLP1/2$_{tetramer}$ in the GDP·AlF_4^- bound state. GDP is represented as red spheres. (**b**) Ribbon schematic showing the superposition of Cj-DLP2 dimer (left), Cj-DLP1$_{\alpha}$ (middle) and Cj-DLP1$_{\beta}$ (right) in the apo and GDP·AlF_4^- bound

states. Apart from the nucleotide-binding pocket, models are similar with RMSD <1 Å. It is speculated that due to high intra-molecular flexibility, crystal packing may force Cj-DLP1/2$_{tetramer}$ into similar conformations irrespective of nucleotide state, therefore masking any potential solution-state conformational change. (**c**) Cartoon schematic showing the superposition of Cj-DLP2$_{\alpha}$ (left) and Cj-DLP1$_{\alpha}$ (right) nucleotide binding pockets in the apo and GDP·AlF_4^- bound states. Side chains are shown for key nucleotide binding and catalytic residues. Nucleotide binding improves stability of the switch 1 loop. No clear density is observed for AlF_4^- in the binding pocket.

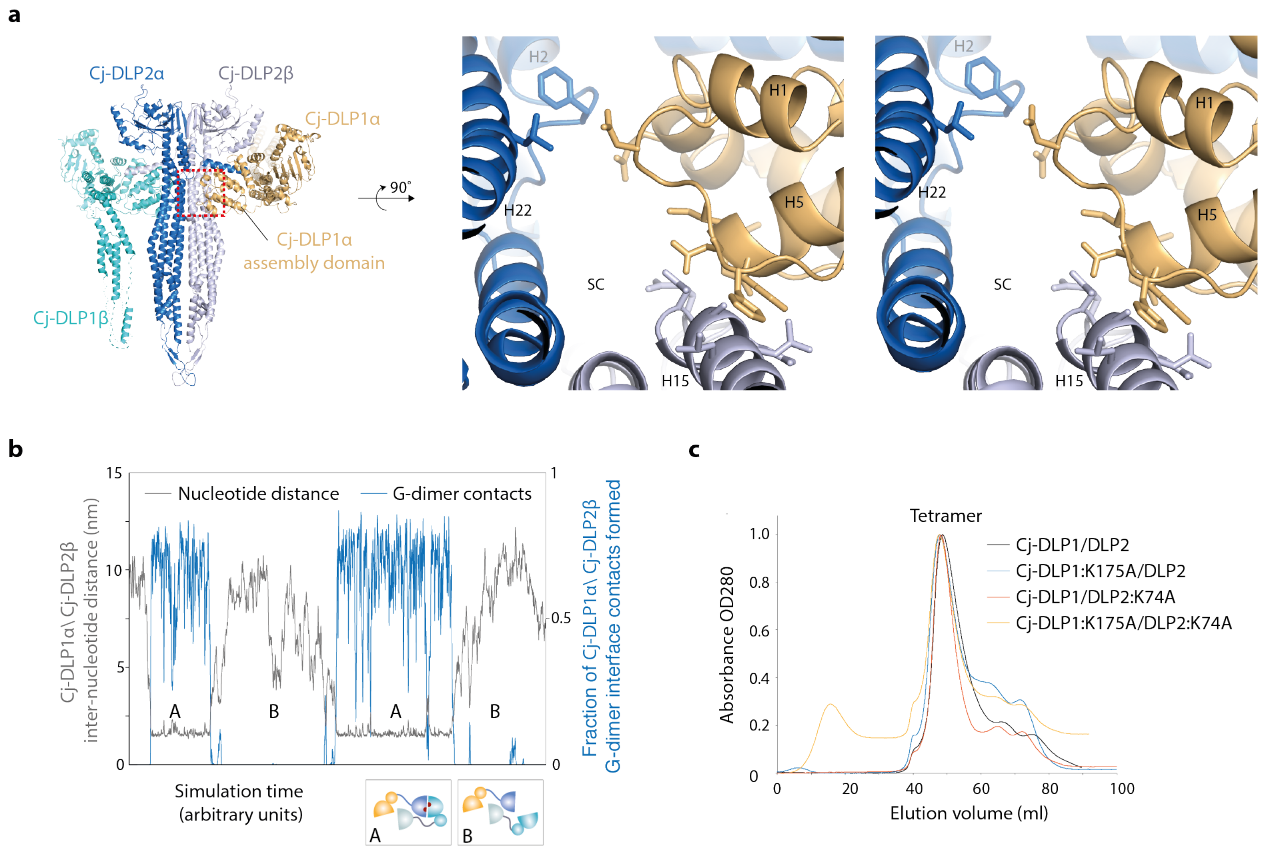

Supplementary Figure 4 | Cj-DLP1/2$_{tetramer}$ oligomerisation and catalysis. (**a**) Cartoon representation focusing on the Cj-DLP1/2$_{tetramer}$ crystal contact between Cj-DLP1$_{AD}$ and Cj-DLP2 neck and trunk interface (left panel, dashed box). Zoomed stereo view of the crystal contact outlined in the dashed box (right panels). Cj-DLP1$_{AD}$ makes limited hydrophobic contact with Helix 22 of Cj-DLP2$_{\alpha}$ and Helix 15 of Cj-DLP2$_{\beta}$. Contact is also weakened by a central solvent channel (SC). (**b**) Excerpt from the molecular dynamics trajectory summarized in Fig. 5d showing inter-nucleotide distance and contact formation between Cj-DLP1$_{\alpha}$ and Cj-DLP2$_{\beta}$ within Cj-DLP1/2$_{tetramer}$. Two cycles of G-dimer formation (inset panel A) and release (inset panel B) are shown. When unbound the heterotypic G-dimer interface typically drifts 10-12 nm apart. (**c**) Gel filtration confirming how Cj-DLP1/2$_{tetramer}$ Walker A point mutations do not affect the oligomeric state.

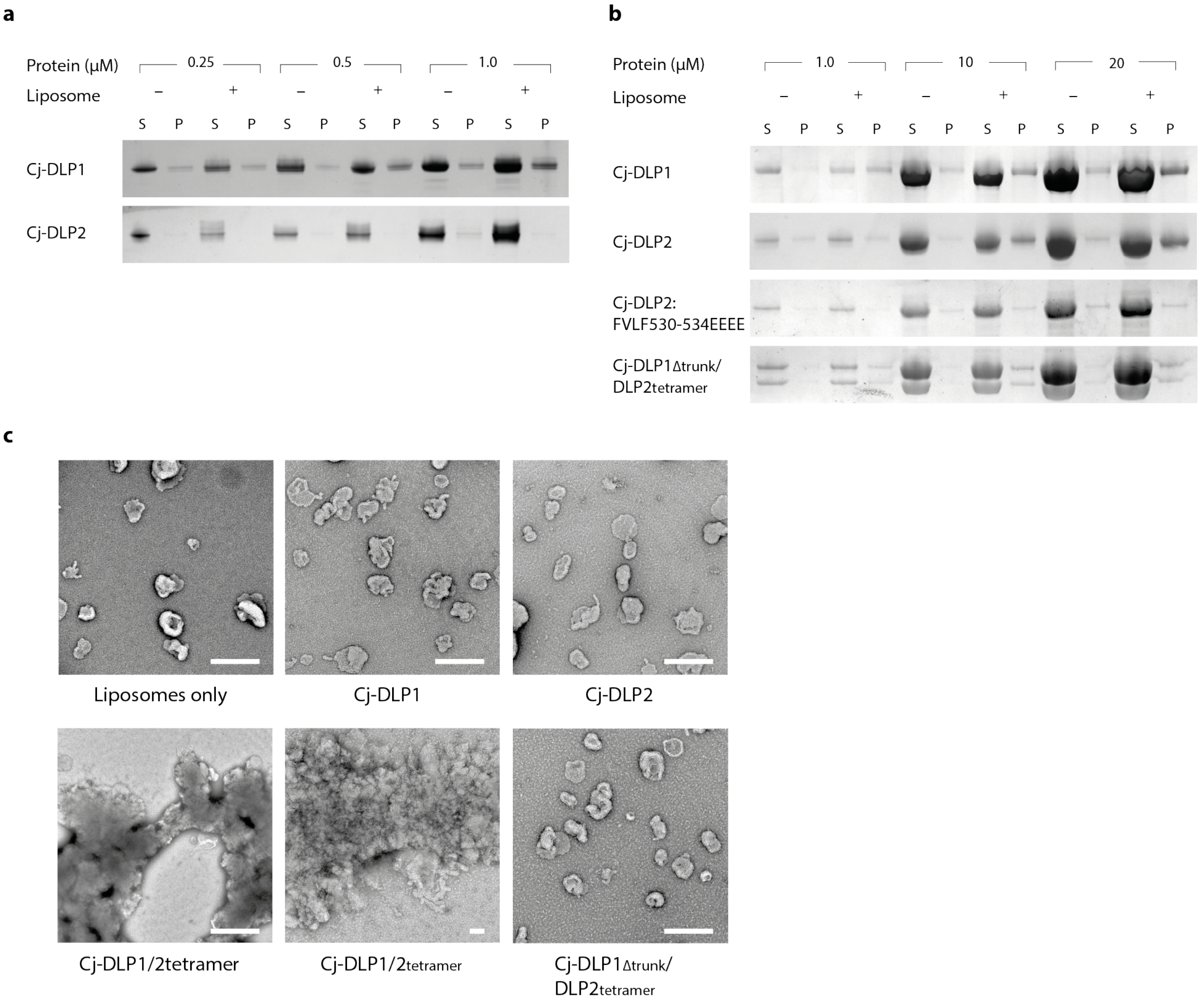

Supplementary Figure 5 | Cj-DLP1 and Cj-DLP2 show modest binding of liposomes *in vitro*. (**a**) Cj-DLP1 and Cj-DLP2 spin assays at concentrations between 0.25-1 μM. (**b**) Cj-DLP1, Cj-DLP2, Cj-DLP2$_{FVLF530\text{-}534EEEE}$, and Cj-DLP1$_{\Delta trunk}$/Cj-DLP2$_{tetramer}$ spin assays at concentrations between 1-20 μM. Note gels were run separately to account for variable silver stain development times. S= supernatant, P= pellet. (**c**) Negative stain EM shows significant Cj-DLP1/2$_{tetramer}$-mediated liposome tethering and possibly fusion at low concentration (0.5 μM). Tethering is not observed with Cj-DLP1 or Cj-DLP2 individually, or with Cj-DLP1$_{\Delta trunk}$/DLP2$_{tetramer}$. Scale bar = 200 nm.

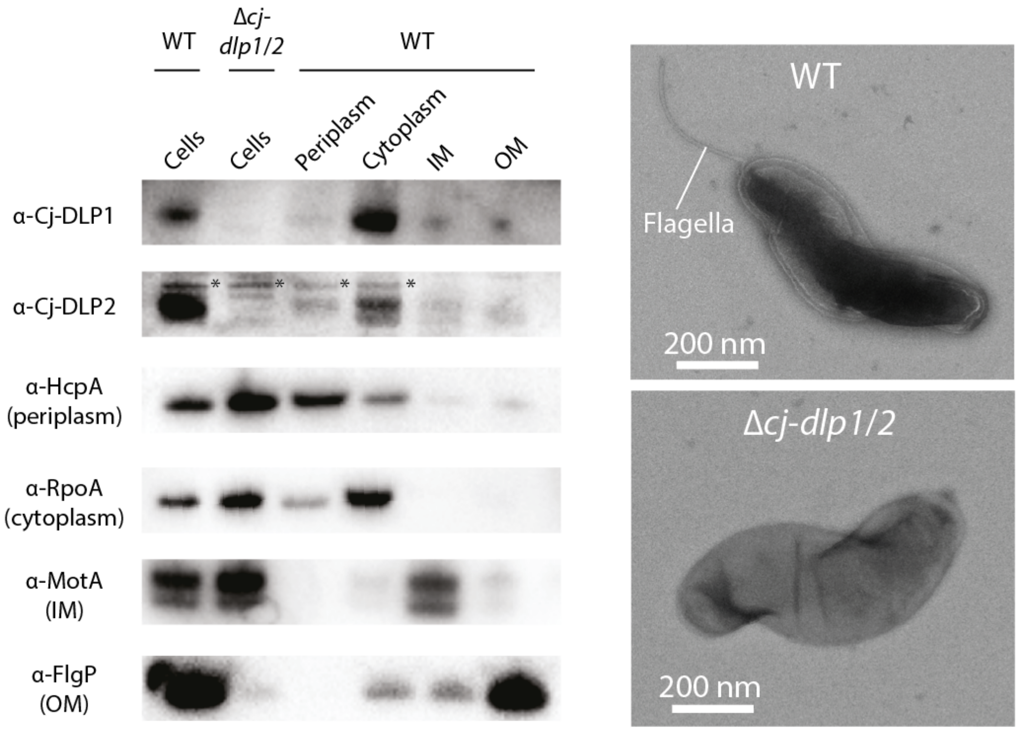

Supplementary Figure 6 | Cj-DLP1 and Cj-DLP2 are expressed in *Campylobacter jejuni*. Both *cj-dlp1* and *cj-dlp2*, and the HcpA-like β-lactamase gene *cj0413* within the *cj-dlp1/2* operon (Fig. 1b) are actively expressed as detected by Western blot against *Campylobacter jejuni* whole cell lysate (left panel). * represents non-specific contaminant for Cj-DLP2 antibody. Fractionation of cellular compartments showed that Cj-DLP1 and Cj-DLP2 localise to the cytoplasm. A chromosomal knockout of *cj-dlp1* and *cj-dlp2* (*Δcj-dlp1/2*) abolished cellular expression. The expression of gene *cj0413* located immediately downstream of *cj-dlp2* (Fig. 1b) was not disturbed in the *Δcj-dlp1/2* strain. Full blots are shown in Supplementary Figure 8. Negative stain EM of *Δcj-dlp1/*2 cells revealed an absence of flagella (right panels) and this was confirmed by a loss of motility phenotype and whole cell cryo-tomography which showed an absence of complete flagella motors (data not shown). No other obvious perturbation to the cell was observed. Here the absence of flagella is speculated to be a pleiotropic consequence of reduced flagella protein FlgP expression in the

Δcj-dlp1/2 strain (left panel) rather than a direct function of Cj-DLP1 and Cj-DLP2. Note that a loss of motility phenotype was also observed in *leoA* knockouts in ETEC [2].

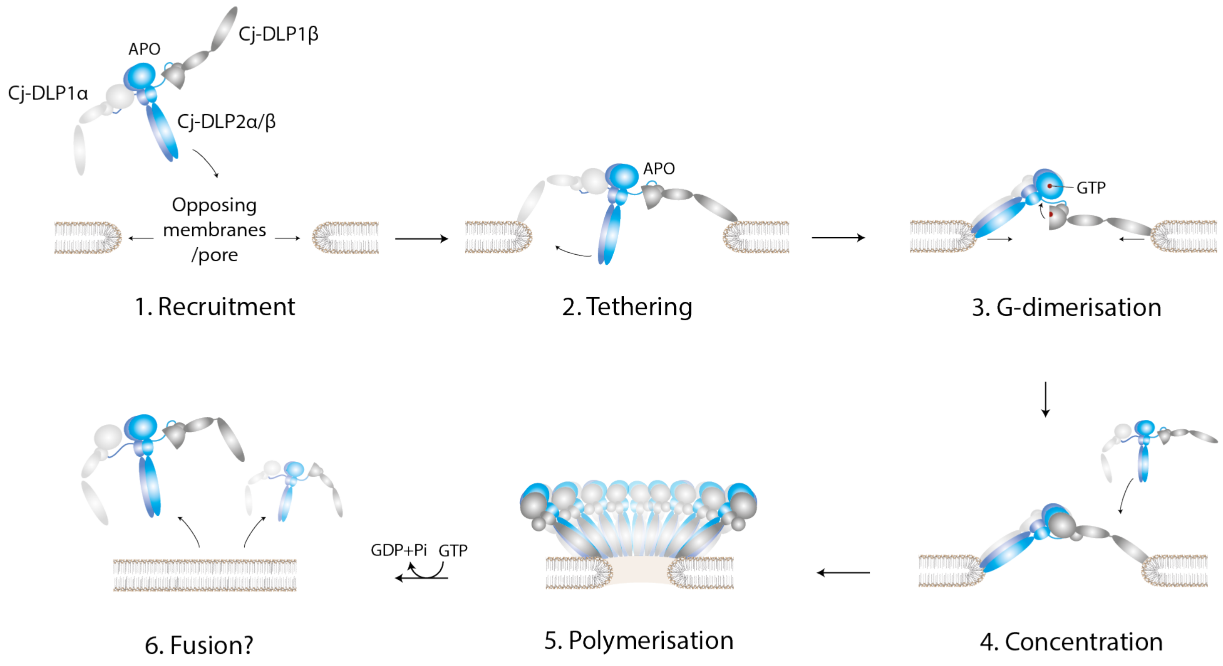

Supplementary Figure 7 | Schematic model for Cj-DLP1/2$_{tetramer}$ membrane tethering on a single membrane. Cj-DLP1/2$_{tetramer}$ architecture facilitates the sensing, recruitment and tethering of opposing membranes that in bacteria likely constitute pores, tears, or regions of high curvature and stress at the inner membrane. Cj-DLP1/2$_{tetramer}$ recruitment to membrane is nucleotide independent. At low concentration, tethering is dependent on Cj-DLP1 with Cj-DLP2 acting as an adaptor to extend the reach of bound Cj-DLP1 subunits. Cj-DLP2 binds to the membrane cooperatively or at higher concentrations. Nucleotide binding induces hetero G-dimerisation between Cj-DLP1 and Cj-DLP2. Local concentration on the membrane surface induces polymerisation and membrane constriction akin to liposome tubulation. The precise role of nucleotide is still to be determined. Nucleotide hydrolysis may be coupled to active fusogenic conformational changes or used to release Cj-DLP1/2$_{tetramer}$ from the membrane.

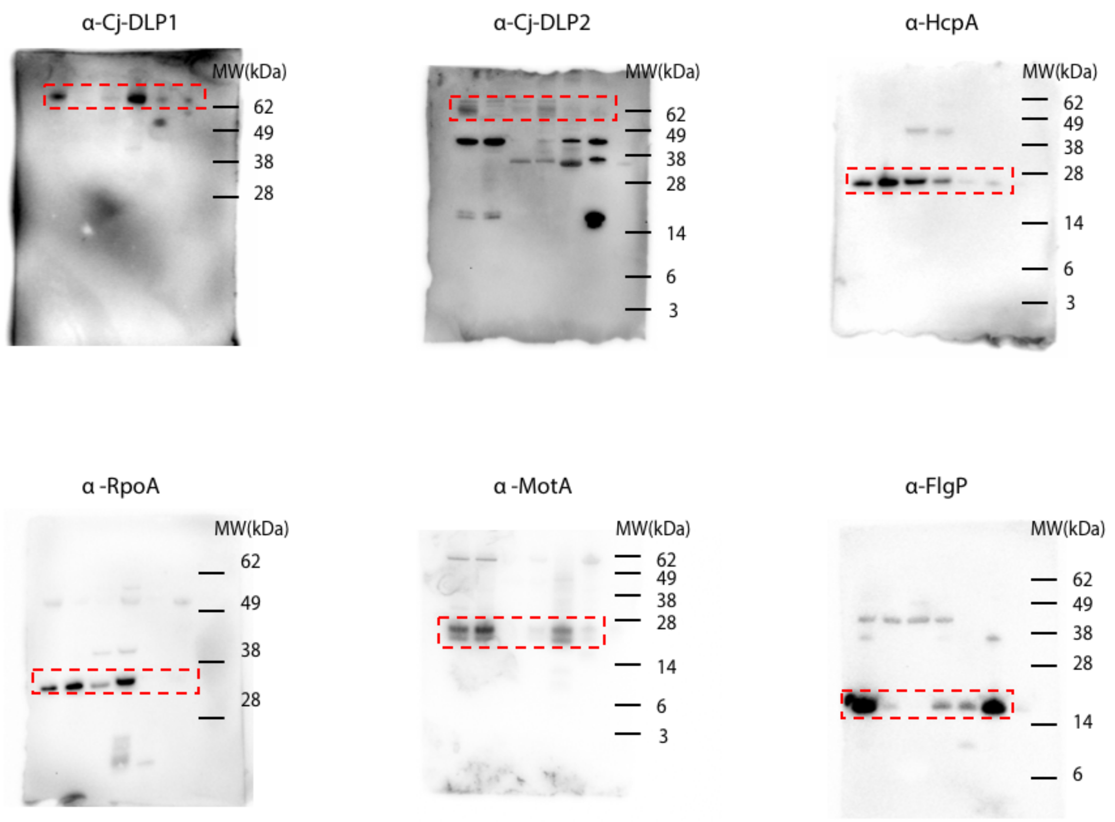

Supplementary Figure 8 | Uncropped western blots shown in Supplementary Figure 6.

Each blot is representative of two independent experiments which yielded similar results.

Primer Name	Sequence	Target Construct Name
pHis17_CJD1_1F	GAAGGAGATATACATATGAAAGAATTATTCCAAAAAATTTGGC	pHis17_cj-dlp1
pHis17_CJD1_2B	GTGGTGGTGAAGCTTATCTGCATTTAAAAGCCCCATTTC	pHis17_cj-dlp1
pOPTM_CJD2_1F	TCTTTTAAATGATTTTATAAAAGCCTATGAAAATACC	pOPTM_cj-dlp2
pOPTM_CJD2_2B	TCGAACTGCGGGTGGCTCCAATCTTCCTTTATACTTTCTCTAACATTTAA	pOPTM_cj-dlp2
pfCJY1-K175A	GCGTGATGAATGCAGGAGCATCAAGTCTTTTAAATGC	pHis17_cj-dlp1:K175A
prCJY1-K175A	GCATTTAAAAGACTTGATGCTCCTGCATTCATCACGC	pHis17_cj-dlp1:K175A
pfCJY2-K74A	GGCAGTTTTCAAGTGGTGCATCAAGTTTGCTAAATTT	pOPTM_cj-dlp2:K74A
prCJY2-K74A	AAATTTAGCAAACTTGATGCACCACTTGAAAACTGCC	pOPTM_cj-dlp2:K74A
pfCJY1-N119	GAAGGAGATATACATATGGATCAAAAAGCTTTATTTCAAA	pHis17_cj-dlp1ΔAD
pRCJY1-C728	GTGGTGGTGAAGCTTATCTGCATTTAAAAGCCCCATT	pHis17_cj-dlp1ΔAD
pR-his17	ATGTATATCTCCTTCTTAAAGTTAAAC	pHis17_cj-dlp1ΔAD
pF-his17	AAGCTTCACCACCACCACCACCACTAAG	pHis17_cj-dlp1ΔAD
T7Fpromoter	TAATACGACTCACTATAGGG	pHis17_cj-dlp1Δtrunk
T7Rpromoter	CCCTATAGTGAGTCGTATTA	pHis17_cj-dlp1Δtrunk
pfCJY1-696	AAGCATGGCACAGACTCGCTGAAAAATCAAGATGCTAATA	pHis17_cj-dlp1Δtrunk
prCJY1-469	CGAGTCTGTGCCATGCTTCTTAATAAGACGATTTTGCATT	pHis17_cj-dlp1Δtrunk
pfCJY2-21NdeI	GGAATTCCATATGTTTGATGATAGTTTTAAAGGGCGTATT	pOPTM_cj-dlp2ΔH1
pR_CJD2_BamHI	CGGGATCCTTAATCTTCCTTTATACTTTCTCTAACAT	pOPTM_cj-dlp2ΔH1
pR-CJY2-347A	TTTACTCATGGCTCTTTCGCTAATGCTTTTAAGCTGTTCT	pOPTM_cj-dlp2Δtrunk-tip
pF-CJY2-402A	AGAGCCATGAGTAAAGAGTTTAAAAAAATCAAAAATG	pOPTM_cj-dlp2Δtrunk-tip
pR-CJY2-508A	TAAAACTCTCGCGCTTAGTTTCGTAGCATTTTCATAATTT	pOPTM_cj-dlp2Δtrunk-tip
pF-CJY2-543A	AGCGCGAGAGTTTTAAATGAATTAAATGTTTATGAGT	pOPTM_cj-dlp2Δtrunk-tip
pfCJY2-FVLF-EEEE	GAGGAAGAAGAGTATCCTAAAAAAAGTGAAATTTATG	pOPTM_cj-dlp2:FVFL530-534EEEE
prCJY2-FVLF-EEEE	ATACTCTTCTTCCTCTTCAGAACTATCAAGCTCATAAAGA	pOPTM_cj-dlp2:FVFL530-534EEEE
pF_PUC19	GGATCCTCTAGAGTCGACCTG	pUC19-cj_dlp1/2-hcp
pR_PUC19	GGTACCGAGCTCGAATTCACT	pUC19-cj_dlp1/2-hcp
pf_operon	TTCGAGCTCGGTACCGTGAAAGAATTATTCCAAAAAATTTGG	pUC19-cj_dlp1/2-hcp
pR_operon	GACTCTAGAGGATCCTTAATAAAGCTCTATATCTTTAGAATT	pUC19-cj_dlp1/2-hcp
pF_Kan	GATAAACCCAGCGAACCATTTGAGGTG	pUC19-cj_Δdlp1/2
pR_Kan	AAGCTTTTTAGACATCTAAATCTAGGT	pUC19-cj_Δdlp1/2
pF_delCJY12	ATGTCTAAAAAGCTTGTTAGAGAAAGTATAAAGGAAGATTAA	pUC19-cj_Δdlp1/2
pR_delCJY12	TTCGCTGGGTTTATCAATTTTTTCTAAAATTTTTAGAGCCTT	pUC19-cj_Δdlp1/2

Supplementary Table 1 | List of primers

Supplementary References

1. Joosten, R.P. et al. A series of PDB related databases for everyday needs. *Nucleic Acids Research* **39**, D411-D419 (2011).
2. Brown, E.A. & Hardwidge, P.R. Biochemical characterization of the enterotoxigenic *Escherichia coli* LeoA protein. *Microbiology* **153**, 3776-3784 (2007).
