# Supplementary material for: Structural basis for membrane tethering by a bacterial dynamin-like pair
Source: Nat Commun. 2018 Aug 21;9:3345. doi: 10.1038/s41467-018-05523-8 (PMC6104087; doi:10.1038/s41467-018-05523-8)
Supplement: Supplementary file 3 — Description of Additional Supplementary Files [file 41467_2018_5523_MOESM3_ESM.pdf]

Description of Additional Supplementary Files

File Name: Supplementary Movie 1
Description: **Molecular dynamics simulation of Cj-DLP1/2$_{tetramer}$ in solution state.** The structure used to build the model of the Cj-DLP1/2$_{tetramer}$ is as shown in Fig. 5c, with G-domain interface contacts additionally included (see Methods). Note that the trunk domains of both Cj-DLP1 molecules have been removed for clarity. The length of Cj-DLP2$_{linker}$ is sufficient for G-domain heterodimerisation, where Cj-DLP1$_{\alpha}$ G-dimerises exclusively with Cj-DLP2$_{\beta}$, and Cj-DLP1$_{\beta}$ exclusively with Cj-DLP2$_{\alpha}$. The alternate conformation in which Cj-DLP1$_{\alpha}$ G-dimerises with Cj-DLP2$_{\alpha}$ was not observed. Since this alternate interface is energetically equivalent in the force field, this means the alternate interface is geometrically occluded. The movement of Cj-DLP1$_{\alpha}$ and Cj-DLP1$_{\beta}$ relative to the central Cj-DLP2 dimer allows distantly opposing membranes to be sensed and bound.
